# Similar complications and outcomes with simultaneous versus staged bilateral total hip arthroplasty with the direct anterior approach: A comparative study

**DOI:** 10.1051/sicotj/2024028

**Published:** 2024-08-22

**Authors:** Christos Koutserimpas, Edouard Rob, Elvire Servien, Sébastien Lustig, Cécile Batailler

**Affiliations:** 1 Orthopaedic Surgery and Sports Medicine Department, Croix-Rousse Hospital, University Hospital 69004 Lyon France; 2 LIBM – EA 7424, Interuniversity Laboratory of Biology of Mobility, Claude Bernard Lyon 1 University 69100 Lyon France; 3 Univ Lyon, Claude Bernard Lyon 1 University, IFSTTAR, LBMC UMR_T9406 69622 Lyon France

**Keywords:** Total hip arthroplasty, Bilateral, Simultaneous procedure, Staged procedure, Direct anterior approach

## Abstract

*Introduction*: Simultaneous bilateral total hip arthroplasty (THA) has demonstrated similar clinical outcomes to staged bilateral THA. However, there is scarce data regarding the early postoperative complications. This study compares simultaneous to staged bilateral THA with the direct anterior approach (DAA) regarding early complications and revision surgeries. *Methods*: This retrospective case-control study included all bilateral THAs, performed by DAA between 2013 and 2021 with a minimum follow-up of 6 months. A total of 264 THAs (132 patients) were identified [simultaneous group (1T): 58 patients; staged group (2T): 74] with a mean follow-up of 54 months. Complications and revisions, clinical outcomes, and days off work were assessed at the last follow-up. Moreover, blood loss was evaluated by the modified method of Mercuriali and Inghilleri. *Results*: Blood loss was higher in the 1T group (1003 mL 1T vs. 740 mL 2T; *p* < 0.001) but there was no significant difference in transfusion rates (5% 1T vs. 3% 2T; *p* = 0.4). There were no complications in 1T, while the complication rate was 5.2% (*n* = 6) in 2T (*p* = 0.012). There were 5 revisions in the 2T group, including 2 debridements with polyethylene exchange and implant retention for early infections, 2 revisions for aseptic loosening in the same patient, and 1 revision due to fracture. Postoperative pain on D3 was equivalent in both groups (4.2 1T vs. 4.3 2T; *p* = 0.79). The improvement in function according to the HHS at 2 months was better in the 1T group, but not significant (36.8 1T vs. 32.9 2T; *p* = 0.05). The total number of days off work was significantly higher in the 2T group (82.6 days vs. 178.8; *p* = 0.025). *Discussion*: Simultaneous bilateral THA with the DAA seems to be a safe procedure, with no risk of increased early postoperative complications when compared to the staged procedure with similar functional outcomes and significantly fewer complications and days off work.

## Introduction

The hip joint represents one of the body’s largest weight-bearing joints, second only to the knee, and is frequently afflicted by osteoarthritis (OA). Hip OA stands out as one of the most common and incapacitating issues impacting older individuals, with 7–8% of the population over 60 having symptomatic hip OA, while up to 25% of them may require bilateral surgery [1–3]. Total hip arthroplasty (THA) has emerged as one of the most successful orthopedic surgeries conducted over the past five decades with high satisfaction rates and improvement in the patient’s quality of life [1, 4, 5].

Bilateral hip osteoarthritis is common, leading to significant long-term risk of requiring contralateral operation among patients undergoing an initial THA [3, 6–8]. Given that patients with contralateral hip osteoarthritis during the initial hip replacement are at an increased risk of subsequent hip surgery [9], it is pertinent to question the optimal strategy for them: either a bilateral THA in a single operating session or two separate sessions.

However, debates persist regarding the choice between 1- or 2-stage (staged) bilateral THA [6, 7]. Opting for 1-bilateral THA offers advantages such as a single anesthesia session and a solitary hospital stay, leading to a shorter overall hospital length of stay (LOS) and cost savings [10, 11]. Several studies have shown similar complication rates between simultaneous and staged bilateral THA [2, 11–15]. The direct anterior approach (DAA) has demonstrated its ability to achieve swifter postoperative recuperation and reduced incidence of complications in contrast to alternative approaches, such as the widely used posterior approach (PA) [16–19]. Moreover, the DAA enables patients to remain in a supine position throughout the procedure, potentially reducing operation duration and eliminating the need for positional adjustments during simultaneous bilateral THA [10].

Some studies are revealing that the complication rates are low when performing bilateral THA by DAA in one stage [10, 16–19]. On the other hand, few studies in the literature have evaluated and compared early complications of bilateral THA in one stage to two stages by DAA [20–23]. The present study aims to evaluate the early complication rate of bilateral THA with the DAA and to compare it to the staged THA.

## Materials and methods

### Population sample

**Figure 1:**
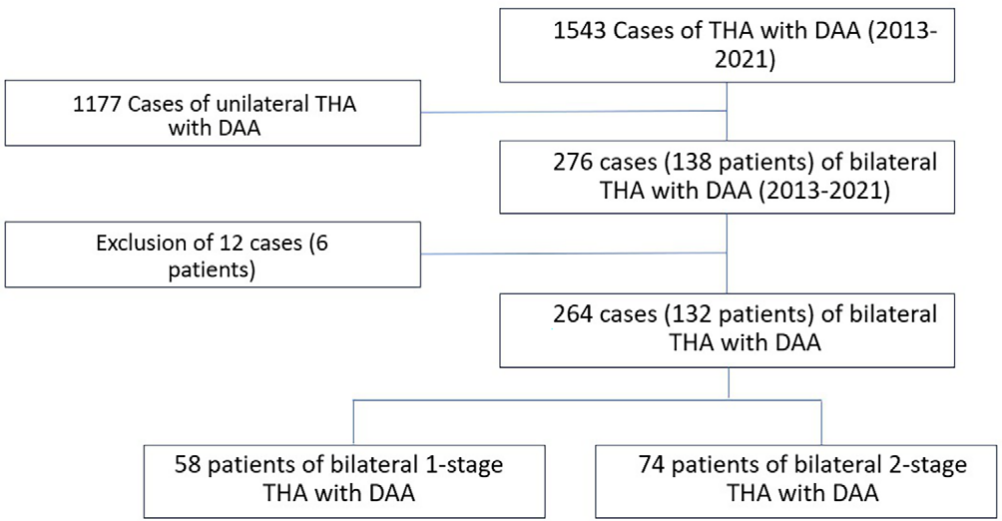
Flow-chart of the patient’s selection. THA: Total Hip Arthroplasty, DAA: Direct anterior approach.

**Figure 2:**
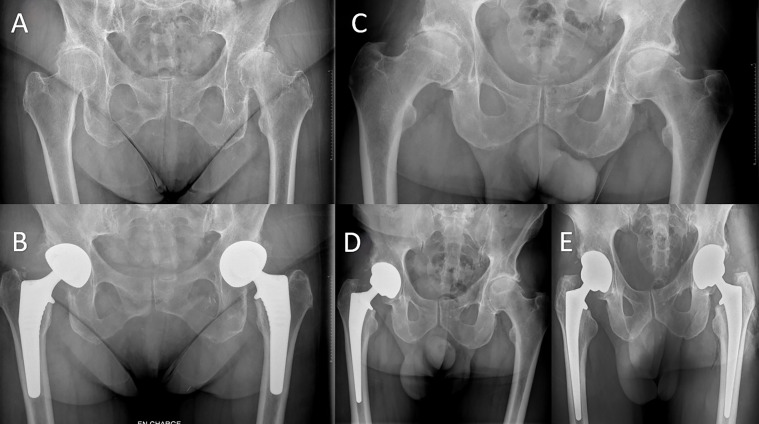
A, B: Presentation of a 1-stage bilateral total hip arthroplasty (THA) with the direct anterior approach (DAA). C–E: Presentation of a 2-stage bilateral THA with the DAA. The time interval in this case between the two surgical interventions was 14 months.

This retrospective single-center study included all patients undergoing bilateral primary THA with DAA from 2013 to 2021. Out of 1453 primary THA patients with the DAA, a total of 138 patients were included. Exclusion criteria encompassed: another lower limb surgery during the initial operation or follow-up, such as knee arthroplasty and THA due to fracture. Six patients had to be excluded because they had had a knee prosthesis. Among the 132 patients who met all the inclusion criteria, 58 underwent bilateral 1-stage (simultaneous) THA (1T) and 74 bilateral 2-stage THA (2T). Figure 1 highlights the patients’ selection, while Figure 2 exhibits examples of the 1-and 2-stage operation. The average final follow-up was 54.05 months, with a minimum of 6 months and a maximum of 100 months.

The patients were matched on the following criteria: age at the time of surgery (the first surgery for the 2-stage group), gender, body mass index (BMI), and the American Society of Anesthesiologists (ASA) physical status. Table 1 summarizes the matching information between the two groups.

**Table 1:** The basic demographic characteristics of the two groups are presented. BMI: Body Mass Index, ASA: American Society of Anesthesiologists. (^*^) Mann–Whitney *U* test. (^#^): Pearson *χ*^2^ test.

	1-Stage group	2-Stage group	*p*-value
Age	61.8 ± 11 [18–78]	64.0 ± 12 [18–83]	0.081^*^
Gender (F/M)	23/58 (39.7%)	45/74 (60.8%)	0.003^#^
BMI (kg/m^2^)	26.4 ± 4.9	26.8 ± 4.5	0.617^*^
ASA score	1.68 ± 0.5	1.77 ± 0.6	0.339^*^

The only difference was gender: the one-stage group comprised 39.7% of females while the two-stage group comprised 60.7% (*p*-value 0.003).

### Surgical technique

All patients in this study were operated on by the same surgeon (SL), using the technique described by Batailler et al: Hueter’s anterior approach [24]. Spinal or general anesthesia was used. The most painful hip was operated on first, both in the 1T and 2T. All implants were uncemented with either a dual mobility cup or a single mobility cup depending on the patient’s age (patients over 70 years of age received a dual mobility component). A redon drain was placed and was removed on the first postoperative day. The patients followed the same Fast Track rehabilitation protocol, including mobilization on the same day of the operation and gradual abandonment of any walking aid within 3 weeks after surgery. Moreover, patient-controlled analgesia was used. A local infiltration of analgesia was used in all cases.

### Data assessment

Demographic data (age, gender, BMI, and ASA score), as well as the preoperative Harris Hip score (HHS), were collected during consultations for anesthesia and surgical planning [25]. At the 2-month follow-up, the HHS was also completed.

The hematocrit was recorded preoperatively and on the first postoperative day (D1). The volume of blood loss was estimated in the operating room and calculated by the modified method of Mercuriali and Inghilleri according to gender with the pre and postoperative hematocrit level, the patient’s weight and the volume of blood, transfused as described in the Appendix [26].

The pain was assessed on the third postoperative day (D3) with the visual analog scale (VAS). During the clinical follow-up at two months, the postoperative appearance of deep vein thrombosis, pulmonary embolism, and infection were also evaluated. The long-term follow-up allowed us to evaluate the time to return to work, surgical revisions of THAs, and deaths.

### Ethical approval

All procedures performed in studies involving human participants were in accordance with the ethical standards of the institutional and/or national research committee and with the 1964 Helsinki Declaration and its later amendments or comparable ethical standards. The Advisory Committee on Research Information Processing in the Field of Health (CCTIRS) approved this study on June 4, 2015, under number 15-430.

### Statistical analysis

For the statistical analysis, the XLs-stat software (2015; Addinsoft) was used. The continuous variables were averaged and reported with standard deviation and interval. The Mann–Whitney nonparametric test was performed to assess the evolution of the HHS. Statistical analysis was performed using the Student *t*-test or Wilcoxon nonparametric test. The categorical variables were compared using the Fisher exact test. Both univariate and multivariate analyses were performed, according to the results, to find predictive factors for the RTS. The potential predictive factors assessed were: age, gender, ASA score, BMI, and HHS. A *p*-value < 0.05 was considered statistically significant in each analysis.

## Results

The average duration between two operations for two-stage bilateral THA was 23.15 months (2–80).

The two studied groups presented ASA scores that did not have a statistical difference [1T; 1.68 (1–3), compared to 2T; 1.77 (1–3) *p*: 0.339]. There was also no difference regarding age [1T; 61.79 years ± 10.09 (15–78), compared to 2T 64.03 years ± 12.0 (18–83)]. No cases of death were recorded.

There was a statistically significant difference in blood loss between the two groups. The average blood loss evaluated intraoperatively was statistically higher in the 1T: (540 mL vs. 337 mL; *p* < .001). Significant higher blood loss in the 1T was also noted with the Mercuriali method: (1003 mL vs. 740 mL; *p* < .001).

Regarding the difference in hematocrit between the preoperative value and the value on D1: the reduction in hematocrit was statistically greater in the 1T (7.26 points vs. 5.56 points; *p* < .001). However, there was no statistically significant difference between the two groups regarding the transfusion rate (5.17% in 1T vs. 2.70% in 2T; *p* = 0.411). A total of 3 patients out of 58 (5.17%) had to be transfused postoperatively in the 1T, compared to 4 out of 74 (5.4%) in the 2T.

We did not note any infections in 1T at any time during the follow-up. Two infections (1.4%) occurred for 2T. In particular, one was recorded 1 month postoperatively and the other at 1 year in two different patients.

No revision surgeries were recorded in the 1T during the follow-up. On the other hand, five revisions (3.4%) were performed in the 2T. In particular, the two infection cases were treated with lavage, debridement, and exchange of the mobile parts of the THA. One case was revised due to an occult intraoperative fracture 3 months after the initial surgery, while another patient required revision surgery for both THAs 1 year after each operation due to aseptic loosening of the femoral component on one side and the acetabular and the femoral component on the other side. No case of dislocation was recorded for both groups.

No cases of deep vein thrombosis were clinically documented in this study. A pulmonary embolism occurred in the 2T (0.7%), documented by pulmonary CT angiography.

The total complication rate was not statistically different between the two groups (5.2% in the 1T, compared to 8.1% in the 2T; *p*: 0.105).

On D3, there was no significant difference regarding pain with the use of VAS (1T: 4.19 ± 1.9, compared to the first stage of the 2T: 4 .31 ± 1.8; *p*: 0.791 and to the second stage of the 2T: 4.28 ± 2.1; *p*: 0.976).

The HHS was significantly worse preoperatively in the 1T (54.59 vs. 59.93; *p*: 0.011), as well as at a two-month follow-up (91.4 vs. 94.99; *p* < 0.001). On the other hand, during the same period, the improvement between the two groups showed a statistically significant better trend in the 1T (36.82 vs. 32.91; *p*: 0.0504).

A total of 18 patients in the 1T and 16 patients in the 2T had active professional lives. We did not find any statistically significant difference concerning the number of days of sick leave before returning to work (1T: 24.6 days ± 41.6 vs. first stage of the 2T: 23.92 ± 88.0; *p*: 0.114; vs. second operation of the 2T: 18.80 ± 2.1; *p*: 0.976). On the other hand, the difference in the number of days of cumulative sick leave was significantly less for the 1T (82.6 days vs. 178.81; *p*: .0245) (Table 2).

**Table 2:** The functional outcomes of the two groups in terms of the Harris Hip Score (HHS), as well as the days off work are presented.

	1-Stage group	2-Stage group	*p*-value
HHS preoperatively	54.6 ± 16	59.9 ± 15	0.011
HHS postoperatively	91.4 ± 8	95.0 ± 10	<0.001
Increase of HSS	37.8 ± 17	32.9 ± 15	0.045
Days off work	75.9 ± 31	1st operation: 82 ± 38	NS
		2nd operation: 70.1 ± 13	
Days off work (in total)	75.9 ± 31	142.7 ± 47	0.028

## Discussion

Bilateral hip osteoarthritis is prevalent, and research indicates that individuals undergoing an initial THA face a significant likelihood of requiring surgery on the opposite hip in the long term [6, 9]. It is noted that whether a bilateral THA performed in a single operating session or two separate sessions would be more advantageous warrants consideration [6, 7]. The present study revealed that the functional outcome assessments indicate comparable results between patients undergoing bilateral simultaneous THA with the DAA and those undergoing the staged THA with the same surgical approach, showing similar scores, albeit with a trend towards improved outcomes with the sequential procedure. However, simultaneous bilateral THA was correlated with notably fewer complications and shorter durations of time off work.

The present study has some limitations. It is retrospective, from a single center and it was focused on the short-term complications. However, it is a comparative study with similar samples and evaluates the outcomes of patients who were managed with the same protocols. Moreover, it offers valuable insights in terms of not only complications but also blood loss, revisions, and functional outcomes.

Kim et al. studied complications in 978 1-stage bilateral THAs [27]. They found an equivalent risk of complications when compared to a unilateral THA population with similar ASA physical status. Nevertheless, it should be noted that there was no description of the used approach or technique in this study [27]. In the present study, the two groups (1-stage and 2-stage THA) had similar ASA scores, age, as well as BMI, as shown in Table 1, while the DAA was used in both groups with the same technique, as well as the same postoperative care and rehabilitation. It is important to specify such details since early outcomes and blood loss could be affected. Weinstein et al. have revealed in a cohort of 43 patients that 1-stage bilateral THA with the DAA had favorable outcomes [12].

Mast et al. in a series of 147 one-stage bilateral THA with DAA estimated that the average blood loss was 578 mL, with 38.8% necessity of transfusion [18]. Although this study did not take into account the ASA’s physical status, it did not record any thromboembolic events, infections, or deaths during the follow-up, while a single intraoperative fracture was noted [18]. Brown et al. studied 44 bilateral DAA THAs in sequential compared to 15 bilateral THAs in 1 stage [8]. They revealed a statistically significant difference in intraoperative blood loss, with an average of 337 mL for the 2-stage surgery vs. 562 mL per surgery for the 1-stage, while Tang et al. recorded mean blood loss of 775 ± 300 mL for 22 patients who underwent bilateral simultaneous THA with DAA [8, 10]. In the present study, the mean intraoperative blood loss for bilateral THA with the DAA was 540 mL which was significantly higher when compared to the 2-stage group (337 mL). Nevertheless, it is of note that the transfusion rates between the 2 groups of the study did not reach statistical significance (5.17% in 1T vs. 2.70% in 2T; *p* = 0.411). On the other hand, Villa et al. found a significantly higher transfusion rate in the 1-stage group: [45.9%, 28/61 (1-stage) vs. 6.3%, 9/143 (2-stage; first operation) vs. 7.0%, 10/143 (2-stage; second operation), *p* < 0.001] [28]. Many factors should be taken into consideration when evaluating transfusion rates, such as the matched groups in terms of age and preoperative hematocrit, the use of autologous RGC, and the sample size [19–21, 24, 29–31]. We also noted significantly higher blood loss in the 1-stage group was also noted with the Mercuriali method: (1003 mL vs. 740 mL *p* < .001), while no other study, to the best of our knowledge, has evaluated blood loss in these cases with this method so far. However, it’s crucial to interpret these findings in context. In the 1-stage THA group, higher blood loss was observed, which can be attributed to the cumulative effect of the two sequential interventions.

Regarding revisions, complications, and infections, the present study did not record any infections in 1T, while two infections (1.4%) occurred for the 2T during the 54.05-month follow-up. Furthermore, no revisions were performed in the 1T, while 5 were recorded in the 2T. The total complication rate was not statistically different between the two groups (5.2% in the 1T, compared to 8.1% in the 2T; *p*: 0.105). Micicoi et al. reported in 327 patients undergoing bilateral hip surgery a similar rate of infections during 1-stage and 2-stage THA [6]. Similar complication rates have been reported in other studies involving minimally invasive DAA and fast-track rehabilitation protocols [15, 16, 32].

Regarding postoperative pain, functional outcomes, and return to work, this study revealed that on D3, there was no significant difference regarding pain with the use of VAS between the 2 groups, while the functional outcomes with the use of HHS seem to be similar, with a tendency to better results with the sequential procedure. Moreover, simultaneous bilateral THA was associated with significantly fewer days off work. Lassik et al. found in a cohort of 408 active Finnish patients operated on for THA between 1999 and 2011 an average of 3 months (10 days to 1 year) before returning to work [33]. We found an average time of 24 days but pain management techniques have evolved over the last decade [34]. Gong et al. revealed a mean VAS score on the D3 similar to the one reported in this study, while Yoshii et al. found a statistically significant improvement for pain at 1 year in the bilateral simultaneous group when compared to the 2-stage group by utilizing the Japanese Orthopedic Association Hip Disease Evaluation Questionnaire [35, 36]. Furthermore, regarding the HSS, Tang et al. found in 22 patients who underwent one-stage bilateral THA an average Harris score of 28 preoperatively and 90 at an average of 16 months postoperatively (8–24 months), while Parcells et al. found a mean Harris score at 3 months of 93.5 in a series of 22 1-stage bilateral THAs using the anterior approach [10, 20]. They did not show a statistically significant difference with their cohort of 22 unilateral THA by DAA [20].

In conclusion, this study by comparing 2 groups with similar ASA scores, age, and BMI has shown that the 1-stage THA with the DAA is a safe procedure since it does not present higher complication rates than the 2-stage operation for patients with hip OA. The functional outcomes appear comparable, as both groups demonstrated similar scores. However, simultaneous bilateral THA was notably linked to fewer complications and reduced total days off work, supporting the actual strategy to offer 1 stage procedure when both hips are symptomatic and the medical condition is appropriate.

## Data Availability

The datasets used and analyzed during the current study are available from the corresponding author on reasonable request.

## Appendix

### Appendix: Total blood loss formulas

Total blood loss was calculated with the following formulas:

Estimated blood volume (mL) = Women: [Body surface area (m^2^)] 2430 Men: [Body surface area (m^2^)] 2530 Body surface area (m^2^) = 0.0235 [height (cm)]0.42246 [weight (kg)]0.51456 And then, to obtain a result with blood loss at a Hct level of 35 percent Total blood loss (mL) = [Total RBC loss (mL)]/0.35.

Uncompensated RBC loss (mL) = [Initial RBC (mL)] [Final RBC (mL)]

Initial RBC = [Estimated blood volume (mL)] [Initial Hct level (%)] at Day –1

Final RBC (mL) = [Estimated blood volume (mL)] [Final Hct level (%)] at Day +1

Compensated RBC loss = [Sum of RBCs received from the various sources of transfusion]

Total RBC loss (mL) = [Uncompensated RBC loss (mL)] + [Compensated RBC loss (mL)]

Estimated blood volume (mL) = Women: [Body surface area (m^2^)] 2430 Men: [Body surface area (m^2^)] 2530 Body surface area (m^2^) = 0.0235 [height (cm)]0.42246 [weight (kg)]0.51456 And then, to obtain a result with blood loss at a Hct level of 35 percent Total blood loss (mL) = [Total RBC loss (mL)]/0.35.
